# COVID-19 and renal involvement: a prospective cohort study assessing the impact of mild SARS-CoV-2 infection on the kidney function of young healthy males

**DOI:** 10.1007/s11255-022-03301-6

**Published:** 2022-07-25

**Authors:** Khalid Al Rumaihi, Kareim Khalafalla, Mohamed Arafa, Arun Nair, Ahmad Al Bishawi, Areen Fino, Faheem Sirtaj, Mohamed Khair Ella, Haitham ElBardisi, Muhammad Abu Khattab, Ahmad Majzoub

**Affiliations:** 1 Department of Urology, Hamad Medical Corporation, 3050 Doha, Qatar; 2 Department of Clinical Urology, Weill Cornell Medicine-Qatar, Doha, Qatar; 3 Department of Andrology, Cairo University, Cairo, Egypt; 4 Department of Medicine, Museaid Hospital, Hamad Medical Corporation, Doha, Qatar; 5 Department of Infectious Diseases, Communicable Disease Center, Hamad Medical Corporation, Doha, Qatar

**Keywords:** COVID-19, Kidney, Renal function, Mild disease, Young patients, Creatinine, Albumin

## Abstract

**Purpose:**

COVID-19 frequently affects the kidneys with symptoms ranging from mild proteinuria to progressive acute kidney injury. This prospective study aimed to assess the short- and long-term impact of asymptomatic and mild COVID-19 on the renal function of healthy young adults, and to determine the correlation between viral load and kidney function among these patients.

**Methods:**

This was a prospective cohort study conducted over a period of 6 months. Patients were followed-up at baseline, and then after 3 and 6 months, respectively. Real-time PCR cycle threshold (CT) was used to determine the viral load and disease activity. Patients were classified into two groups with either asymptomatic COVID-19 or mild pneumonia. The assessment parameters were variables that could directly or indirectly relate to the renal function.

**Results:**

A total of 48 patients were included and evaluated. The majority of patients (62.5%) had asymptomatic COVID-19 disease. Patients with mild pneumonia had significantly higher serum creatinine (SCr) at the time of COVID-19 diagnosis (beta = 12.836, 95% CI = 2.405–23.268, *P* = 0.019), after 3 months (beta = 14.345, 95% CI = 1.149–27.542, *P* = 0.035), and after 6 months (beta = 14.100, 95% CI = 0.730–27.470, *P* = 0.040) compared to asymptomatic patients. Mild pneumonia was also significantly associated with lower serum albumin level at the time of COVID-19 diagnosis (beta = – 6.317, 95% CI = – 9.448–− 3.185, *P* < 0.001).

**Conclusion:**

Mild COVID-19 is associated with mild renal involvement without AKI. Changes in the renal function appear to be related to reduced creatinine clearance and possible albumin leakage in the acute phase of the disease. The reduction in creatinine clearance is not predicted by viral load, and it appears to be a long-term effect of the disease that can last for at least 6 months.

## Introduction

Severe acute respiratory syndrome coronavirus 2 (SARS-CoV-2) has spread globally, and was associated with over 250 million confirmed cases of coronavirus disease 2019 (COVID-19) and over 5 million deaths as of December 19, 2021 [1, 2]. The manifestations of the disease can vary from mild and self-limiting for most patients into a severe condition with respiratory or multiorgan failure [3]. The patient age and comorbid conditions are important predictors of the clinical course and disease severity [4]. Common symptoms of mild COVID-19 include fever, dry cough, dyspnea, fatigue, sore throat, and diarrhea. Nonetheless, the nervous system, gastrointestinal system, cardiovascular system, and the kidneys are reportedly involved [5, 6].

COVID-19 frequently affects the kidneys with symptoms ranging from mild proteinuria to progressive acute kidney injury (AKI) requiring renal replacement therapy [7]. The incidence of AKI can range between 15 and 25% based on the disease severity, and is associated with higher mortality rates of the disease [8, 9]. This renal impairment is characterized by elevated serum creatinine (SCr), blood urea nitrogen (BUN), proteinuria, and hematuria. Indeed, the virus tropism to the kidney, and serum creatinine and BUN are reportedly elevated in greater than 10% of the patients and in over 30% of severe cases [10, 11]. Moreover, about half of patients with the disease had experienced proteinuria and hematuria, along with the detection of the SARS-CoV-2 RNA in the urine samples of the patients [12–14].

SARS-CoV-2 impact the kidney through the angiotensin-converting enzyme 2 (ACE2), cellular transmembrane serine protease 2 (TMPRSS2), and cathepsin L (CTSL). These enzymes are substantially expressed in the kidneys and were found to be essential for the virus entry [10]. The ACE2 RNA expressions in the kidneys are about 100-fold greater than the lungs, and the co-expression of ACE2 and TMPRSS has been found to be significantly high in proximal straight tubule cells and podocytes, indicating a favorable localization environment for the virus [12, 15]. The exact mechanism of COVID-19 associated AKI is not fully understood, although it is suggested to be the result of cytokine storm and the direct cytopathic effect of the virus [10]. Acute tubular necrosis is the most common clinical manifestation, though the tubulointerstitial, vascular, and glomerular systems of the kidneys can all be affected [16].

The first case of COVID-19 was declared in Qatar on February 29th, 2020, a 36-year-old Qatari male returning back to Doha [17]. The national governmental and healthcare systems ever since are reacting to this unprecedented situation. Most of the current cases are mild owing to mass vaccination in the state; however, the weekly rate of new cases remains at 6% which is almost double than the global rate [2, 18]. Several studies evaluated the extrapulmonary impact of the mild form of the disease on different organ system, yet the studies that assessed the urinary system are limited. Furthermore, studies originating from different regions around the world suggested that patients with diabetes, hypertension, cardiopulmonary, and pre-existing renal disease are at a higher risk of complications and mortality [19–22]. Male gender and renal dysfunction are reportedly correlated with higher risk of mortality, suggesting to closely monitor renal function particularly among males to effectively control the disease [23–25]. Gender differences in habitual exposures (smoking), health status and immune system have been proposed as potential reasons for the higher mortality risk among males [26]. Nonetheless, an increase in gene expression of ACE2 and TMPRSS2 proteins in males may be considered a more plausible association with this observation [27, 28].

Literature on AKI among non-comorbid patients with mild COVID-19 is scarce, and there is lack of studies on the instant and long-term renal complications associated with mild forms of the disease. This prospective study aimed to assess the short- and long-term impact of asymptomatic and mild COVID-19 on the renal function of healthy young adults, and to determine the correlation between viral load and kidney function among these patients.

## Methods

### Study design and participants

This was a prospective cohort study conducted at the department of urology at Hamad medical corporation (HMC), Doha, Qatar, over a period of 6 months. Patients with COVID-19 identified by a positive polymerase chain reaction (PCR) result were screened for eligibility. The inclusion criteria were young fit male patients aged between 18 and 55 years with asymptomatic or mild COVID-19. The reason of selecting healthy young patients was to determine whether SARS-CoV-2 can lead to renal dysfunction in the absence of predisposing factors. Furthermore, only men were included in this study as it was part of a larger project which assessed the impact of COVID-19 infection on various male reproductive parameters. Patients with comorbid conditions including diabetes mellitus, hypertension, dyslipidemia, cardiovascular diseases, anemia, and pre-existing renal disease were excluded. Patients with moderate to severe COVID-19 or who were hospitalized due to a COVID-19 related complication were also excluded. The study was approved by the institutional review board at HMC (protocol No. MRC-01–20-347), and all methods were performed in accordance to the institutional guidelines and regulations. All patients had to agree to participate and signed informed consent before being enrolled in the study.

### Procedures and outcomes

Patients were followed-up at the time of COVID-19 diagnosis (the baseline), and then after 3 and 6 months, respectively. Real-time PCR cycle threshold (CT) was used to determine the viral load and disease activity. Higher concentration of viral genetic material and activity were determined at low CT [29]. A lower CT was also assumed to be associated with a higher risk of infectivity. Blood investigations were done at baseline, 3-months, and 6-month to determine the impact of mild SARS-CoV-2 infection on the kidneys. The assessment parameters were variables that could directly or indirectly relate to the renal function and included complete blood count (CBC), SCr (umol/L), BUN (mmol/L), electrolytes, calcium (nmol/L), bicarbonate (HCO_3_^−^) (mmol/L), serum albumin (mmol/L), and fasting blood glucose level (FBG) (mmol/L). All laboratory measures were performed by an autoanalyzer (Hitachi 747 autoanalyzer; Tokyo, Japan). Clinical data were also obtained, and patients were classified into two groups according to the NIH COVID-19 treatment guidelines [30]. The first group included asymptomatic COVID-19 patients who could be identified exclusively by their positive PCR testing. The second group on the other hand included patients with mild pneumonia as confirmed by chest radiography, and who were hemodynamically stable according to their vital signs and oxygen saturation. The primary outcome measure was the short- and long-term impact of COVID-19 on kidney function of healthy individuals. The secondary outcome measure was to determine the correlation between viral load and renal involvement.

### Statistical analysis

Statistical analyses were performed using IBM statistical package for the social sciences (SPSS, version 25). Continuous variables were expressed by their mean and standard deviation. Categorical variables were expressed by their frequencies and percentage. The normal distribution of variables was tested using histogram and Shapiro–Wilk test. The clinical and laboratory parameters were compared in the univariate analysis between the two groups using independent sample *T* test and Pearson correlation coefficient. A multivariable analysis was conducted utilizing repeated measures analysis of variance (ANOVA) to determine the short- and long-term impact of COVID-19 at the three-time interval of assessment (baseline, 3-month, and 6-month). The results were reported by their estimated marginal means and unadjusted beta with 95% confidence interval. *P* values of 0.05 and less were considered statistically significant with an acceptable margin of error of 5%.

## Results

### Baseline characteristics of patients

A total of 70 patients were included, among which 48 completed all evaluation and were included in the analysis. The mean age of patients was 35.10 (± 5.63), the average CT of COVID-19 was 23.39 (± 5.23), and the majority of patients (62.5%) had asymptomatic disease. The mean levels of BUN and SCr were 3.91 (± 1.21) and 81.10 (± 14.60), respectively. The mean of serum albumin was 39.83 (± 4.42), and the mean of venous bicarbonate was 25.15 (± 1.57). The full baseline characteristics of the patients are shown in Table 1.

**Table 1:** Baseline characteristics of patients

Variable	Mean (± SD) or frequency (%)
Age	35.10 (5.63)
COVID-19 severity indicators	
CT	23.39 (5.23)
Asymptomatic	30 (62.5)
Mild pneumonia	18 (37.5)
CBC	
White blood cells (WBCs)	6.79 (2.28)
Red blood cells (RBCs)	5.26 (0.43)
Hemoglobin	15.10 (1.10)
Hematocrit	44.63 (3.21)
Platelets	280.42 (123.80)
Renal function	
BUN	3.91 (1.21)
SCr	81.10 (14.60)
Serum electrolytes and minerals	
Na^+^	137.25 (2.48)
K^+^	4.48 (0.65)
Cl^−^	100.21 (2.69)
Ca^2+^	2.34 (0.12)
HCO_3_^−^	25.15 (1.57)
Serum albumin	39.83 (4.42)
FBG	6.27 (1.63)

### Univariate association of disease severity with clinical and laboratory parameters

The clinical presentation of COVID-19 was significantly associated with patients’ age. Patients with mild pneumonia had significantly older age compared to asymptomatic patients (37.61 vs. 33.60, *P* = 0.015). A significant association between disease severity and renal function was found. Mild pneumonia had a significant borderline association with higher levels of SCr compared to asymptomatic disease (90.56 vs. 82.20, *P* = 0.054). The BUN levels were also higher with mild pneumonia, though the difference between the groups was not statistically significant (4.13 vs. 3.78, *P* = 0.410). The level of serum albumin was significantly lower with mild pneumonia (36.50 vs. 41.83, *P* < 0.001). Patients with mild pneumonia had also significantly lower blood levels of sodium and potassium (*P* = 0.024 and < 0.001, respectively). The univariate association of COVID-19 severity and clinical parameters of the patients are presented in Table 2.

**Table 2:** Univariate association of COVID-19 severity and clinical parameters

Variable	Patients with asymptomatic COVID-19 (*N* = 30) Mean (± SD)	Patients with mild COVID-19 pneumonia (*N* = 18) Mean (± SD)	*P* value
Age	33.60 (5.84)	37.61 (4.33)	0.015*
CBC			
White blood cells (WBCs)	7.04 (2.26)	6.37 (2.32) 5.25 (0.58)	0.335
Red blood cells (RBCs)	5.26 (0.31)	14.85 (1.30)	0.929
Hemoglobin	15.21 (0.91)	44.12 (4.01)	0.266
Hematocrit	44.94 (2.65)	255.06	0.396
Platelets	295.63 (85.07)	(170.13)	0.276
Renal function			
BUN	3.78 (0.92)	4.13 (1.59)	0.410
SCr	82.20 (10.73)	90.56 (18.63)	0.054**
Serum electrolytes and minerals			
Na^+^	137.87 (92.22)	136.22 (2.60)	0.024*
K^+^	4.77 (0.54)	4.02 (0.53)	< 0.001*
Cl^−^	100.70 (2.31)	99.39 (3.13)	0.103
Ca^2+^	2.37 (0.10)	2.29 (0.15)	0.060
HCO_3_^−^	25.33 (1.63)	24.83 (1.47)	0.291
Serum albumin	41.83 (3.20)	36.50 (4.20)	< 0.001*
FBG	5.90 (1.04)	6.89 (2.20)	0.086

*Statistically significant

**Borderline statistical significance

A significant weak inverse correlation between viral load as determined by COVID-19 CT and serum albumin was found (r = – 0.317, *P* = 0.028). Viral load was also significantly correlated with red blood cells (RBCs) (r = -0.329, *P* = 0.022) and hematocrit (*r* = − 0.293, *P* = 0.043). No significant negative or positive correlations between the viral load and SCr or BUN were found. The correlation between viral load and clinical parameters are reported in Table 3.

**Table 3:** Correlation between viral load as determined by COVID-19 CT and clinical parameters

Variable	Pearson correlation coefficient (*r*)	*P* value
Age	0.222	0.129
CBC		
White blood cells (WBCs)	0.018	0.904
Red blood cells (RBCs)	– 0.329	0.022*
Hemoglobin	– 0.266	0.068
Hematocrit	– 0.293	0.043*
Platelets	0.178	0.225
Renal function		
BUN SCr	– 0.062 – 0.117	0.676 0.430
Serum electrolytes and minerals		
Na^+^	– 0.173	0.240
K^+^	– 0.061	0.696
Cl^−^	– 0.174	0.236
Ca^2+^	– 0.007	0.962
HCO_3_^−^	0.068	0.648
Serum albumin	– 0.317	0.028*
FBG	– 0.161	0.275

*Statistically significant

### Multivariable repeated measures ANOVA of SCr, BUN, and serum albumin

**Fig. 1:**
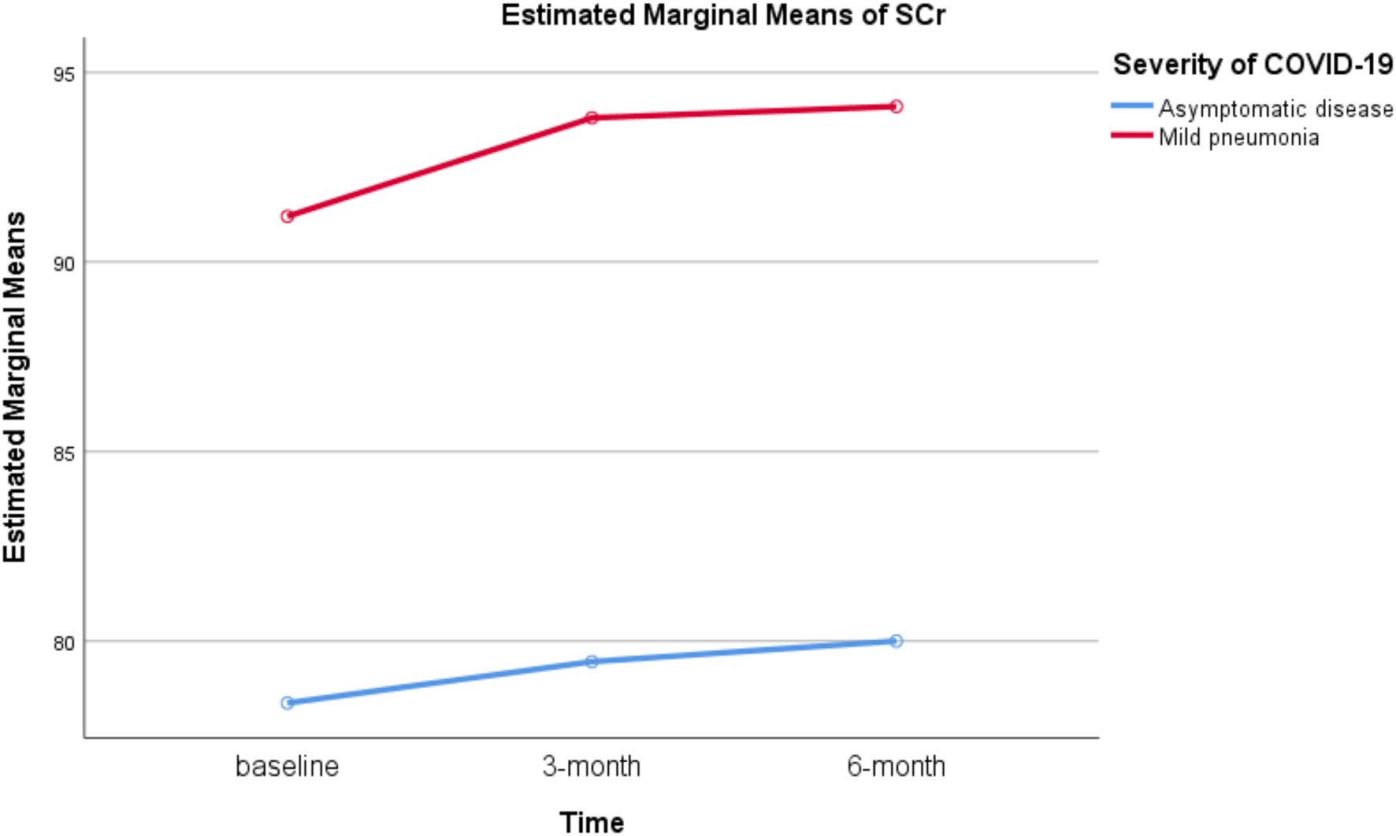
Estimated marginal means of repeated measures of SCr

**Fig. 2:**
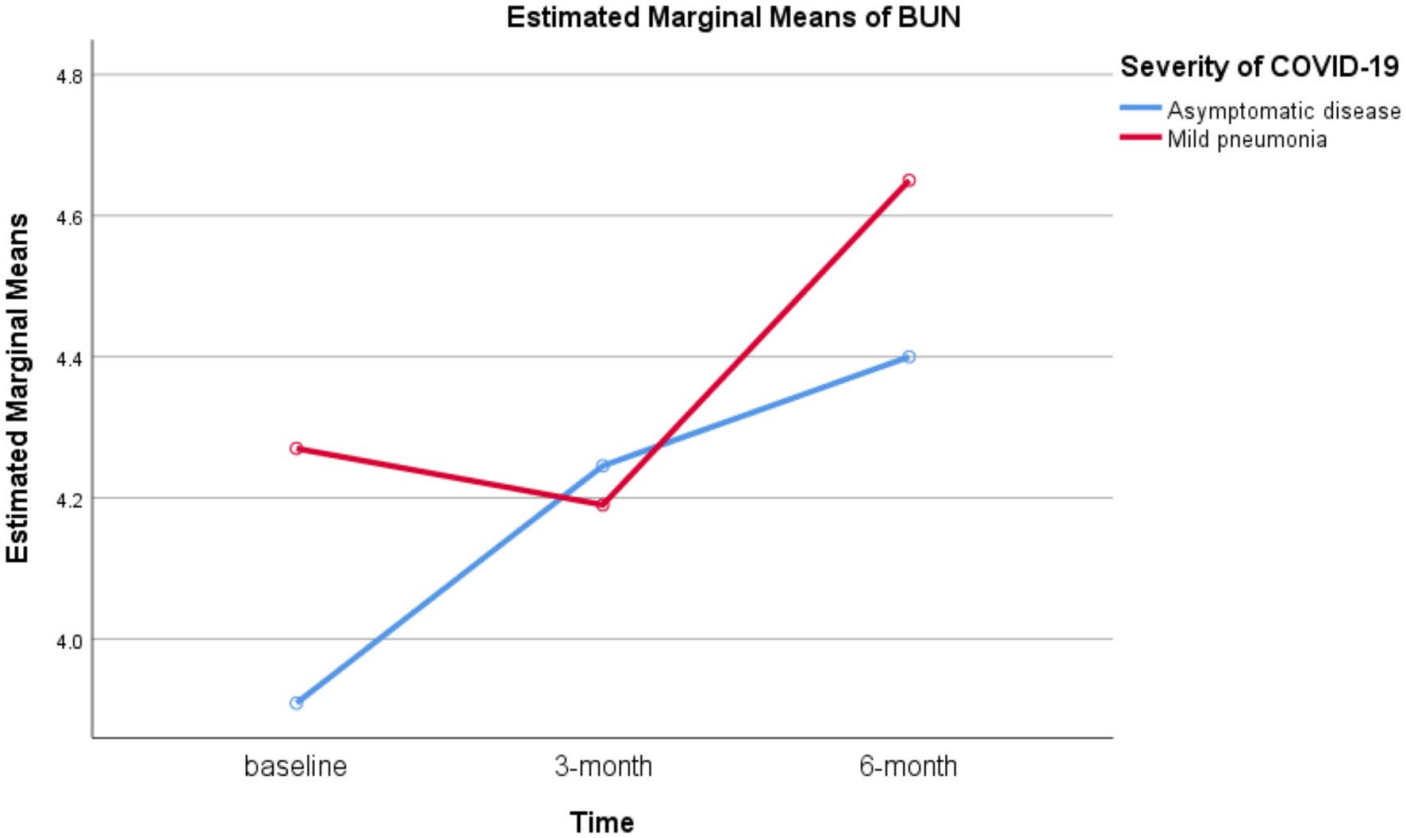
Estimated marginal means of repeated measures of BUN

Patients with mild pneumonia had significantly higher estimated marginal mean of SCr compared to asymptomatic patients (93.033 vs. 79.273, *P* = 0.023). The estimated marginal means of BUN were 4.370 and 4.185 for patients with mild pneumonia and asymptomatic disease, respectively (*P* = 0.647). Figures 1 and 2 present the estimated marginal means of SCr and BUN.

A significant positive association between disease severity and SCr was found on repeated measures. Patients with mild pneumonia had significantly higher SCr at the time of COVID-19 diagnosis (beta = 12.836, 95% CI = 2.405–23.268, *P* = 0.019), after 3 months (beta = 14.345, 95% CI = 1.149–27.542, *P* = 0.035), and after 6 months (beta = 14.100, 95% CI = 0.730–27.470, *P* = 0.040) compared to asymptomatic patients. A significant negative association between disease severity and serum albumin was found. Mild pneumonia was significantly associated with lower serum albumin level at the time of COVID-19 diagnosis (beta = – 6.317, 95% CI = – 9.448 to – 3.185, *P* < 0.001). Disease severity was not significantly associated with serum albumin levels at 3- and 6-month. No significant association was also found between disease severity and the three repeated measures of BUN. The parameter estimates of repeated measures ANOVA are shown Table 4.

**Table 4:** Parameter estimates of repeated measures ANOVA

Variable	Unadjusted beta*	95% confidence interval	*P* value
SCr			
At baseline	12.836	2.405–23.268	0.019**
At 3-month	14.345	1.149–27.542	0.035**
At 6-month	14.100	0.730–27.470	0.040**
BUN			
At baseline	0.361	– 0.832–1.553	0.534
At 3-month	– 0.055	– 1.316–1.205	0.928
At 6-month	0.250	– 0.501–1.001	0.494
Serum albumin			
At baseline	– 6.317	– 9.448–-3.185	< 0.001**
At 3-month	0.200	– 1.929–2.329	0.847
At 6-month	– 0.900	– 3.096–1.296	0.403

*Parameter: mild COVID-19 pneumonia versus asymptomatic disease

**Statistically significant

## Discussion

Previous literature reported that renal involvement is common with COVID-19 and revealed that AKI is highly observed in patients with underlying kidney disease [31–33]. The present study determined the short- and long-term impact of COVID-19 on the renal function of young, healthy and fit patients with mild SARS-CoV-2 infection. It also determined the correlation of viral load with kidney function. We found a significant impact of mild COVID-19 on the renal function as determined by significantly higher levels of SCr and lower levels of serum albumin at the time of diagnosis of the infection. Repeated measures also showed a persistent impact of the disease on the renal function up to 6 months post-infection. No significant associations between the viral load and kidney function were determined.

Mild forms of COVID-19 appear to have a short- and long-term impact on the kidneys of young healthy individuals without pre-existing renal dysfunction or comorbid conditions. We found significantly higher SCr that is associated with lower glomerular filtration rate (GFR) in patients with mild COVID-19 pneumonia compared to asymptomatic patients at the time of diagnosis. Although the higher SCr remained within the normal range, our findings suggest that COVID-19 may be associated with a reduced kidney function in the absence of AKI. Our results are consistent with other findings that determined mild renal function impairment without any evidence of AKI in patients with mild COVID-19 [34]. The findings of the current study add to the literature that renal involvement with COVID-19 can occur in young and previously healthy individuals and without a pre-existing renal disease.

There is scarcity of data in the literature about the long-term effect of COVID-19 on the kidneys. Our results showed that the initial impact of SARS-CoV-2 on the kidney is persistent for at least 6 months, as we found higher SCr levels in patients who had mild COVID-19 pneumonia at 3-month and 6-month post-initial diagnosis. The long-term reduction in renal function of patients with mild COVID-19 pneumonia was also mild and may appear clinically not significant. Nevertheless, it is believed that slight changes in creatinine clearance importantly predict all-cause mortality, and creatinine clearance has a negative correlation with all-cause death in COVID-19 patients with GFR below 150 mL/min/1.73 m^2^ [34, 35].

Although BUN is a more sensitive indictor of kidney function than SCr [36], we did not find a significant difference in the estimated marginal means of BUN between the two study groups. This could be the result of adequate hydration that is recommended within nonpharmacological measures for all patients with COVID-19 [37]. In fact, BUN is a non-specific indicator of kidney function, and hydration is associated with lower levels of BUN in patients with preserved renal function [36, 38].

Previous literature reported renal protein leakage and albuminuria with severe COVID-19 [39–42]. We found significantly lower serum albumin in patients with mild COVID-19 pneumonia. This could be associated with possible protein leakage and albuminuria. SARS-CoV-2 can directly infect the renal parenchyma and induce renal damage, which may be associated with a spectrum of renal manifestation ranging from mild protein leakage to collapsing glomerulopathy [43–45]. Our findings suggest that albuminuria is only a short-term complication of mild COVID-19, as our multivariable repeated measures showed minor fluctuations of serum albumin at 3- and 6-month post-infection that were statistically not significant. Nonetheless, the current study did not evaluate directly albuminuria to provide conclusive results.

The pattern of COVID-19 renal involvement and its mild impact on the SCr and possibly GFR on one hand, and protein leakage on the other hand is suggested to be related to the pathogenesis of SAR-CoV-2 on the kidneys and its consequences. COVID-19 may be associated with renal damage due to cytopathic injury resulting from direct invasion, or through systemic inflammatory responses. Most symptomatic infections are associated with inflammatory responses, however, not all infections can directly invade the kidney [46, 47]. Our findings suggest that mild COVID-19 is not associated with acute tubular necrosis, and that short-term albumin leakage could be the result of glomerulonephritis.

Järhult et al. determined the impact of viral load and viremia on extrapulmonary organ dysfunction and mortality [48]. They confirmed that patients with more viral RNA in the blood (RNAemia) required more renal replacement therapy and had higher morality. The findings of the current study did not show any significant association between viral load and renal function. This can be explained by the fact that mild forms of COVID-19 are associated with lower viral load, which is unlikely to correlate significantly with extrapulmonary organ dysfunction [49].

### Implications for practice

Pertaining to the relative uniqueness and novelty of COVID-19, much remain to be learned regarding its effect on various organ systems and the resulting influence on morbidity and mortality. Extrapulmonary involvement of COVID-19 is linked to increased risk of renal impairment. The long-term impact of renal involvement of COVID-19 on the risk of development of chronic kidney disease and mortality is yet to be addressed. The findings of the present study warrant additional routine assessment to the kidney function of patients with mild COVID-19 who do not require inpatient care.

### Strengths and limitations

This study has several strengths. It is the first study to determine the impact of mild COVID-19 on the renal function of young, healthy, fit, and non-comorbid patients. Unlike other studies with retrospective or cross-sectional design that could not provide temporality, the prospective design of this study provided better evidence with causal relationship about COVID-19 renal involvement. Moreover, the analysis of repeated measures allowed to determine persistent COVID-19 renal consequences for at least 6 months. On the other hand, the limitations of the study include a small sample size, although the number of patients appears to be acceptable as long-term studies involving healthy volunteers are usually associated with high rates of withdrawal and lost to follow-up. The study also did not assess albuminuria and other urine indicators of renal function. Despite that the patients in both groups had serum albumin within the normal range, albumin levels were significantly lower in patients with mild pneumonia, which is assumed to be the result of proteinuria. Further research is suggested in this context to assess the extent of proteinuria in young healthy individuals with mild forms of COVID-19. Finally, the study included young, healthy and fit males only, therefore, the external validity of our findings is limited, and the results cannot be generalized to all patients with mild COVID-19.

## Conclusion

Mild COVID-19 is associated with mild renal involvement without AKI. Changes in the renal function appear to be related to reduced creatinine clearance and possible albumin leakage in the acute phase of the disease. The reduction in creatinine clearance is not predicted by viral load, and it appears to be a long-term effect of the disease that can last for at least 6 months. Future work will involve a larger cohort to prospectively assess the impact of mild COVID-19 on the urinary indicators of renal function including albuminuria and hematuria.
